# Effects of remote ischemic conditioning on conditioned pain modulation and cardiac autonomic modulation in women with knee osteoarthritis: placebo-controlled randomized clinical trial protocol

**DOI:** 10.1186/s13063-023-07527-2

**Published:** 2023-08-07

**Authors:** Taíse Mendes Biral, Allysiê Priscilla de Souza Cavina, Eduardo Pizzo Junior, Carlos Alberto Toledo Teixeira Filho, Franciele Marques Vanderlei

**Affiliations:** 1 Postgraduate Program in Movement Sciences, São Paulo State University (Unesp), School of Technology and Sciences, Presidente Prudente, SP Brazil; 2 Postgraduate Program in Physiotherapy, São Paulo State University (Unesp), School of Technology and Sciences, Presidente Prudente, SP Brazil; 3 Department of Physiotherapy, São Paulo State University (Unesp), School of Technology and Sciences, Presidente Prudente, SP Brazil

**Keywords:** Ischemic preconditioning, Therapeutic occlusion, Conditioned pain modulation, Heart rate variability, Autonomic nervous system, Chronic pain, Osteoarthritis, Randomized controlled trial

## Abstract

**Background:**

It is estimated that over 240 million people worldwide have osteoarthritis, which is a major contributor to chronic pain and central changes in pain processing, including endogenous pain modulation. The autonomic nervous system plays a crucial role in the pain regulatory process. One of the main mechanisms of remote ischemic conditioning is neuronal signaling from the preconditioned extremity to the heart. This study aims to analyze the acute effect of remote ischemic conditioning on local pain, conditioned pain modulation, and cardiac autonomic control in women with knee osteoarthritis and to see if there is a correlation between them.

**Methods:**

Women more than 50 years with knee osteoarthritis diagnosed according to the American College of Rheumatology criteria in the postmenopausal period will be considered eligible. The study will have blind randomization, be placebo-controlled, and be balanced in a 1:1 ratio. The total of 44 participants will be divided into two groups (22 participants per group): (i) remote ischemic conditioning and (ii) placebo remote ischemic conditioning. Protocol consisting of four cycles of total ischemia, followed immediately by four cycles of 5 min of vascular reperfusion, totaling 40 min. The primary outcomes in the protocol are conditioned pain modulation, which has the pressure pain threshold (kgf/cm^2^) as its primary outcome measure, and cardiac autonomic modulation, which has the indices found in heart rate variability as its primary outcome measure. Comparisons will be performed using generalized linear mixed models fitted to the data. For correlation, the Pearson or Spearman test will be used depending on the normality of the data. All analyses will assume a significance level of *p* < 0.05.

**Discussion:**

It is believed that the results of this study will present a new perspective on the interaction between the pain processing system and the cardiovascular system; they will provide the professional and the patient with a greater guarantee of cardiovascular safety in the use of the intervention; it will provide knowledge about acute responses and this will allow future chronic intervention strategies that aim to be used in the clinical environment, inserted in the multimodal approach, for the treatment of osteoarthritis of the knee.

**Trial registration:**

ClinicalTrials.gov NCT05059652. Registered on 30 August 2021. Last update on 28 March 2023.

**Supplementary Information:**

The online version contains supplementary material available at 10.1186/s13063-023-07527-2.

## Introduction

Early prevention and treatment of musculoskeletal disorders and targeting risk factors for associated chronic diseases can play an important role [1]. However, a greater understanding of why musculoskeletal conditions increase the risk of chronic disease is needed. As in the case of osteoarthritis, which appears to increase the risk of developing cardiovascular disease [1].

It is estimated that more than 240 million people worldwide have symptomatic osteoarthritis and among the risk factors for knee osteoarthritis are advanced age and female sex [2]. In addition, individuals with osteoarthritis have lower levels of physical activity [2], which is a major contributor to chronic pain and central changes in pain processing [3].

The pain processing system includes endogenous pain modulation, characterized by the ability of the central nervous system (CNS) to modulate nociceptive input from peripheral tissues as it ascends to the spinal cord, brainstem, and brain [4]. This modulation can lead to an increase or inhibition of pain perception. Furthermore, a dysfunctional modulation may be part of the pathophysiology of chronic pain [4].

Endogenous pain modulation can be investigated through the conditioned pain modulation (CPM) test, which is characterized by the reduction of pain perception caused by intense pain in a remote area of the body and represents the outcome of descending pain inhibitory mechanisms [5, 6]. In summary, among the functional impairments [1] and CNS mechanisms [2] presented by patients with osteoarthritis, the presence of pain due to less efficient endogenous inhibition can be highlighted.

Furthermore, the autonomic nervous system (ANS) plays a crucial role in modulating perception, functional interaction, and pain regulation [7, 8]. The interaction between ANS and pain perception has been shown to be an important contributor to the pain regulatory process [9, 10]. Heart rate variability (HRV) is a reliable non-invasive way to assess ANS modulation. Several chronic pain conditions are being associated with alterations in the ANS function, assessed through HRV indices [11–14].

In summary, the systems involved in autonomic control are strictly linked to those involved in pain perception, and, therefore, HRV can be considered an index of ANS reactivity to nociceptive stimulation [15]. Patients with chronic pain, such as osteoarthritis, have reduced HRV and baroreflex sensitivity due to changes in efferent sympathetic and parasympathetic cardiac activity, which alter the balance to a prevalence of sympathetic tone related to catecholamine release [16, 17].

HRV is also related to the endogenous modulation of pain, a relevant factor in the development and maintenance of pain [18]. Furthermore, endogenous pain modulation and HRV are linked in both the presence and absence of chronic pain. Thus, HRV has several advantages in studies that investigate the physiological response to nociceptive stimulation [19–21].

According to a recent systematic review by Forte et al. [22], as HRV appears to be impaired in several chronic pain conditions that can worsen the quality of life, future researchers and clinicians may benefit from using HRV to assess the effectiveness of treatments in pain management in clinical populations [22].

Therefore, the effect of chronic pain presented by individuals who have osteoarthritis may provide adaptation in the autonomic regulation of the cardiovascular system. Taking this into account, treatment approaches that generate changes in pain processing behavior and cardiac autonomic modulation need to be further explored.

An approach that has been used to improve muscle performance and that is widely used in the management of cardiovascular disorders because it is considered an intrinsic mechanism that has the ability to protect tissues against the deleterious effects of ischemia–reperfusion is remote ischemic conditioning (RIC) [23]; however, little is known about the effect of this intervention for pain management [24] and its repercussion on the CPM and cardiac autonomic control systems. There is no evidence that RIC causes systemic hypoalgesia, so these gaps provide a rationale for future research.

The RIC is characterized by the application of brief periods of circulatory occlusion (ischemia) and reperfusion of a limb through inflation and deflation of a pressure cuff [25]. Thielmann et al. [26] demonstrated reduced myocardial damage from RIC, including improvement in clinical outcomes in patients undergoing cardiac surgery and in those with myocardial infarction undergoing emergency coronary artery stent implantation [26].

One of the main mechanisms of RIC appears to be neuronal signaling from the preconditioned extremity to the heart [27]. Current data reveal that there are interactions of neuronal and humoral pathways and activation of peripheral sensory nerves, resulting in a release of cardioprotective factors that circulate to target organs via the bloodstream [28–30]. In this way, the ANS can be modified using the RIC, which, in turn, can be proven by the HRV indices.

Given the ease of administration of the intervention, in addition to its low cost and not being an invasive method, RIC can be investigated as a possible multimodal analgesic strategy [24]. However, further studies are needed on the impact of different RIC strategies, elucidation of mechanisms, safety profiles, and cost-effectiveness. Furthermore, understanding the immediate cardiovascular benefits of RIC will support the development of strategies in the primary and secondary prevention of cardiovascular disease [27].

Taking into account that the RIC can result in an improvement in cardiac autonomic modulation through the activation of neuronal pathways and that this activation can reflect in a better management of the patient’s pain, the following gaps were raised: Is the RIC capable of improving local pain after its application in women with osteoarthritis? Does RIC cause changes in CPM systems and cardiac autonomic modulation? Is there a correlation between CPM and cardiac autonomic modulation?

From these gaps, two hypotheses will be tested. First, that RIC is able to improve cardiac autonomic modulation and consequently improve CPM in women with knee osteoarthritis. Second, that there is a correlation between CPM and cardiac autonomic modulation in women with knee osteoarthritis. Therefore, the primary objective of the study will be to analyze the acute effect of RIC on local pain, CPM, and cardiac autonomic control systems in women with knee osteoarthritis. The secondary objective will be to observe if there is a correlation between CPM and cardiac autonomic modulation in women with knee osteoarthritis.

Information of this nature is important to guide therapists on aspects of intervention related to CPM and cardiac autonomic modulation, about reducing pain and improving autonomic control, and in this way, it provides guiding knowledge for future long-term studies on other chronic diseases and cardiovascular events.

## Methods

### Trial design

This is a randomized, parallel, placebo-controlled, double-blind, exploratory, 1:1 randomized clinical trial in one of two study groups, namely: RIC or placebo.

The study protocol follows the SPIRIT 2013 checklist (Standard Protocol Items: Recommendations for International Trials) [31] (Supplementary File 1) and the TIDieR (Template for Intervention Description and Replication) [32], so that the information and quality of reports of interventions are well described [32].

### Eligibility criteria

Women with knee osteoarthritis aged between 50 and 80 years will be included in the study if they have unilateral or bilateral knee osteoarthritis [33–35] diagnosed according to American College of Rheumatology criteria [36] which are: criteria for clinical and radiographic diagnosis (having knee pain, osteophytes and having at least one of the three items—age greater than 50 years; stiffness lasting less than 30 min and/or crepitus) or criteria for clinical diagnosis (pain in the knee and having at least three of the six items — age greater than 50 years; stiffness lasting less than 30 min; crepitus; bone enlargement; bone tenderness; no palpable heat) will be considered eligible if they are in the postmenopausal period and not present systemic rheumatic diseases (fibromyalgia, rheumatoid arthritis and systemic lupus erythematosus); congestive heart failure, peripheral vascular disease, systolic blood pressure greater than 160 or less than 100 mmHg or diastolic blood pressure greater than 100 mmHg, deep vein thrombosis, a history of acute myocardial infarction and stroke; diabetes mellitus; respiratory disease; a history of knee surgery; will not be included, as well as participants who have high levels of physical activity assessed by the Habitual Physical Activity Questionnaire [37, 38], are alcoholics, smokers, use drugs that influence cardiac autonomic modulation (beta-blockers) and have one or more predisposing risk factors for thromboembolism [39].

**Fig. 1:**
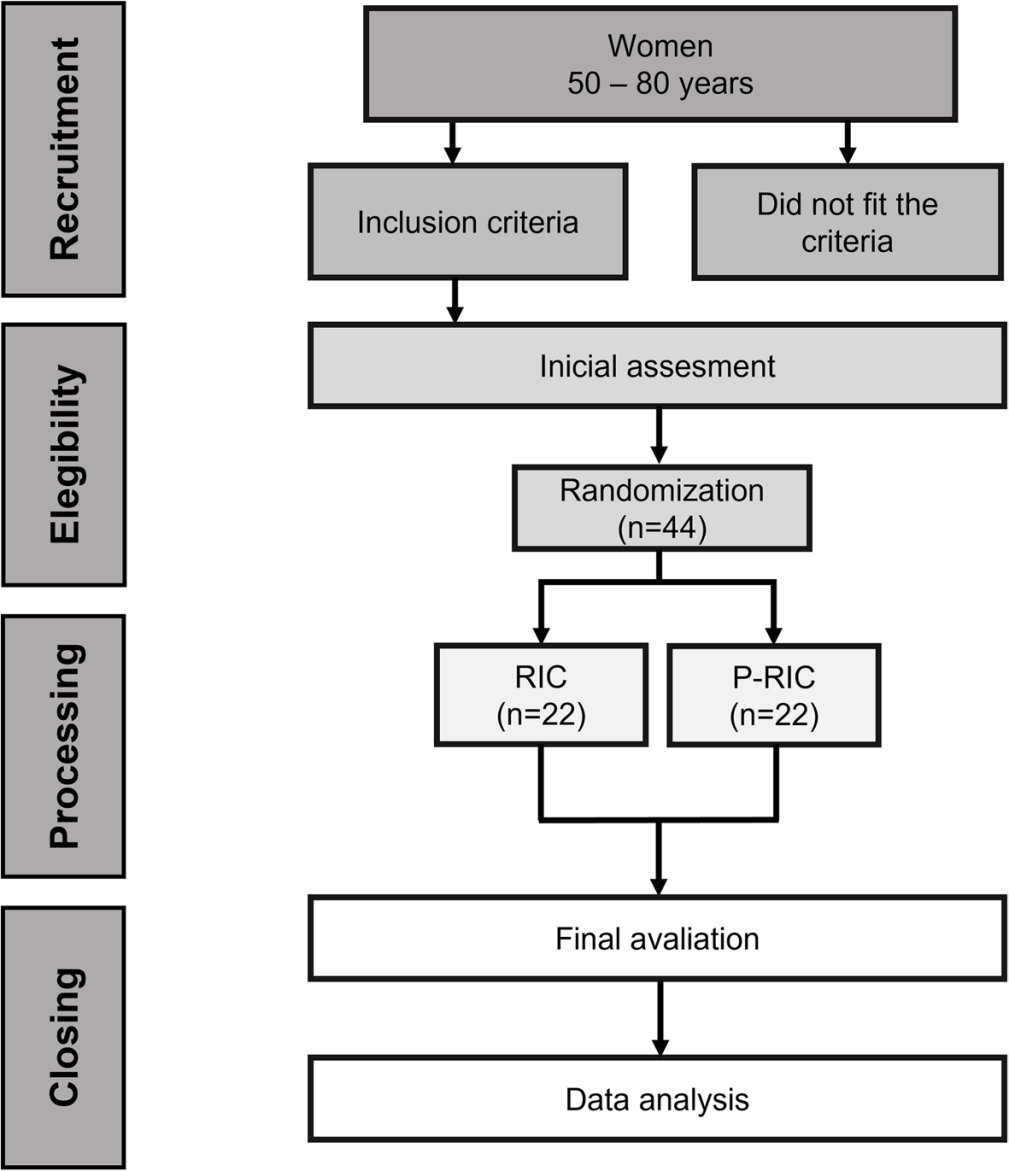
Flowchart of the study design. RIC, remote ischemic conditioning; P-RIC, placebo remote ischemic conditioning

Participants who, in the 24 h before the test, used anti-inflammatory drugs or analgesics, performed physical activity, consumed alcohol, performed therapeutic treatments for pain relief, had errors in capturing the heart rate beat by beat (RR intervals), and wished to withdraw from the study will be excluded. In addition, participants may choose to stop performing the intervention for any reason. The flowchart of the study design and the composition of the groups is illustrated in Fig. 1.

### Recruitment

Participants will be recruited through advertising folders on the institution’s premises, health centers, and social networks. These procedures are recommended by Treweek et al. [40] as strategies to improve the recruitment of participants.

All participants will undergo an initial telephone screening to determine eligibility for inclusion in the study. The following data will be obtained as part of the screening: age, self-reported weight, brief health history including symptoms of knee osteoarthritis (knee joint pain and stiffness), patient comorbidities, and medications in use. Afterwards, the participants will answer the Habitual Physical Activity Questionnaire [38, 39] that assesses the level of physical activity. Those who claim to have knee pain and meet the study inclusion criteria will be invited to remain in the study. The blood pressure eligibility criteria will be personally measured to confirm the pressure measurement reported by the participant over the phone.

Initial assessment data will also be used for sample characterization purposes. If eligible and willing to participate in the study, a time will be scheduled for the participant to attend the Sports Physiotherapy Laboratory. To increase the participants’ adherence to the intervention, telephone contact will be made daily to encourage them to remain in the study, and thus avoid losses.

All participants will go through an informed consent process that will include the delivery of written information about the need and the overall benefit of the study, followed by a discussion with a researcher. This discussion will include verifying their understanding of the benefits and risks of participation and ensuring that participants accept that treatment will be randomly allocated, regardless of any personal preferences they may have. If the participant agrees to participate, a consent form is signed (the consent form template is in the  supplementary materials).

The consent form does not ask whether participants would like to be contacted in the future about research participation. It is worth noting that this study will not perform laboratory evaluation and storage of biological specimens for genetic or molecular analysis at this time or for future use.

### Randomization and blinding

Participants who provide written informed consent will be randomly allocated to either placebo RIC or RIC. The randomization sequence will be elaborated using software (Microsoft Office Excel 2007) and will be placed in sequential numbering in opaque and sealed envelopes. A researcher who will not have contact with the participants and evaluators will generate the allocation sequence (assign the participants to the interventions). Evaluators, participants, and data analysts will be blinded to the allocation of groups. At the end of the survey, to disseminate the results to the participants, the allocation group may be revealed.

There is a study trial management group made up of the study researchers that meets weekly to monitor the progress of the study. Any changes to the protocol will be notified to the ethics committee, an update will be made to the clinical trial registry and will be communicated to study participants.

### Study design

Data collection will be carried out at Sports Physiotherapy Laboratory at São Paulo State University (Unesp), School of Technology and Sciences, Presidente Prudente, São Paulo, Brazil. All procedures will be performed under standardized conditions (temperature: 21–23°C; relative humidity: 40–60%).

Each participant will attend the laboratory on two days separated by one week. On the first day they will be evaluated for anthropometric characteristics, using a scale (Tanita BC 554, Iron Man/Inner, Arlington Heights Illinois, USA) and a stadiometer (Sany—American Medical do Brasil, São Paulo, Brazil) and, later, the body mass index (BMI) will be calculated. Blood pressure measurements will also be performed [41] and the Knee Injury and Osteoarthritis Outcome Score (KOOS) questionnaire [42] will be applied. After these initial procedures, a 10 min rest will be performed and, in the sequence, the evaluation of total occlusion pressure (TOP) will be performed. After that, the initial CPM test will be performed, followed by the RIC, and finally the CPM test again. RR intervals will be captured from the initial 10 min of rest to the end of the complete CPM protocol.

**Fig. 2:**
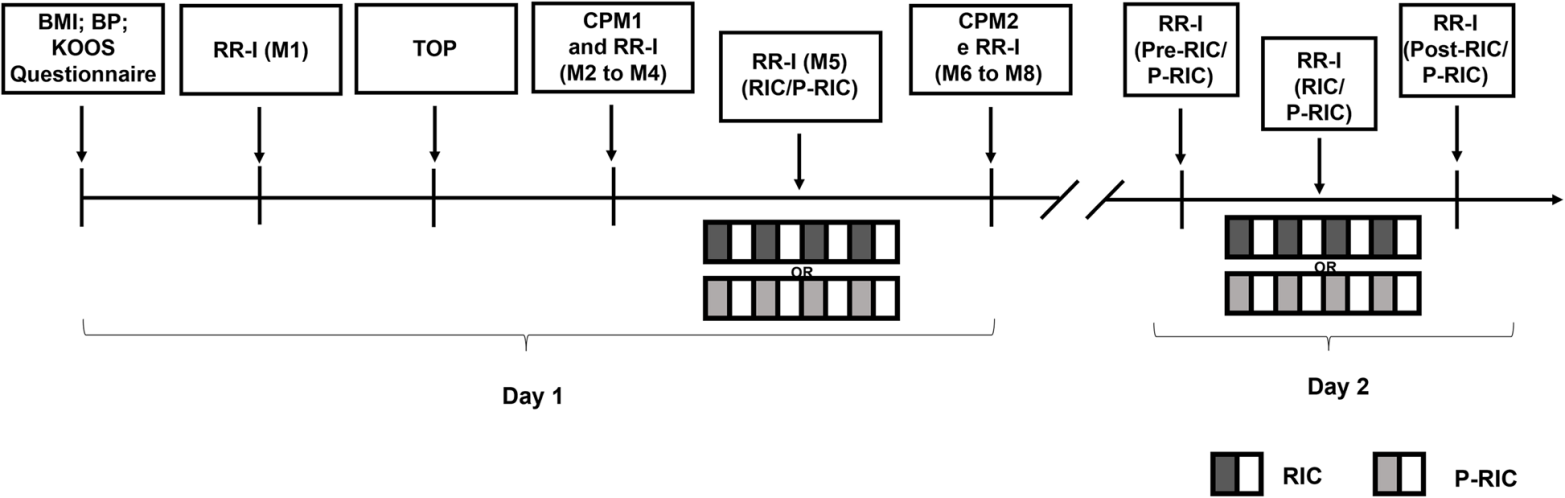
Study design. BMI, body mass index; BP, blood pressure; RR-I, RR intervals; M, moment; TOP, total occlusion pressure; CPM1, initial conditioned pain modulation; CPM2, final conditioned pain modulation; RIC, remote ischemic conditioning; P-RIC, placebo remote ischemic conditioning

On the second day, only the RR intervals will be captured before the RIC (15 min at rest), during, and after (15min at the end) the RIC. The study design is outlined in Fig. 2.

### Interventions

In both study groups, the TOP will be determined: first, participants will be asked to remain at rest in the supine position for 10 min in an acclimatized room (21–23°C) and silent [43, 44]. To determine the TOP, a vascular Doppler (DF-7000 V; Medpej, Ribeirão Preto, São Paulo, Brazil) will be used, which will be positioned over the posterior tibial artery (located in the middle distance between the medial malleolus of the tibia and the Achilles tendon) to capture the auscultatory pulse [45].

A cuff (velcro; 12.5 cm wide and 84 cm long and inflatable chamber 7 cm wide and 52 cm long, Cardiomed, Curitiba, Paraná, Brazil) will be positioned in the region of the inguinal fold with the inflatable region of the cuff in the medial portion of the thigh covering the femoral artery [46] of the lower limb with the greatest complaint of knee pain [45, 46] and then it will be progressively inflated waiting 30 s every 50 mmHg and completely deflating and pausing for 10 s, and 40 mmHg will be added to the initial 50 mmHg (total of 90 mmHg).

The sequence will be repeated, always adding the 40 mmHg until the auscultatory pulse of the tibial artery is completely interrupted. Then wait 30 s and slowly deflate until the auscultatory pulse returns. The TOP will be defined when the lowest pressure capable of completely obstructing blood flow is reached [45, 47–49]. A wider cuff was chosen, as it has been proven that the width of the cuff has a great influence on the pressure needed to achieve full restriction of blood flow [49]. The intervention and placebo groups will be different as described below.

### Remote ischemic conditioning

**Fig. 3:**
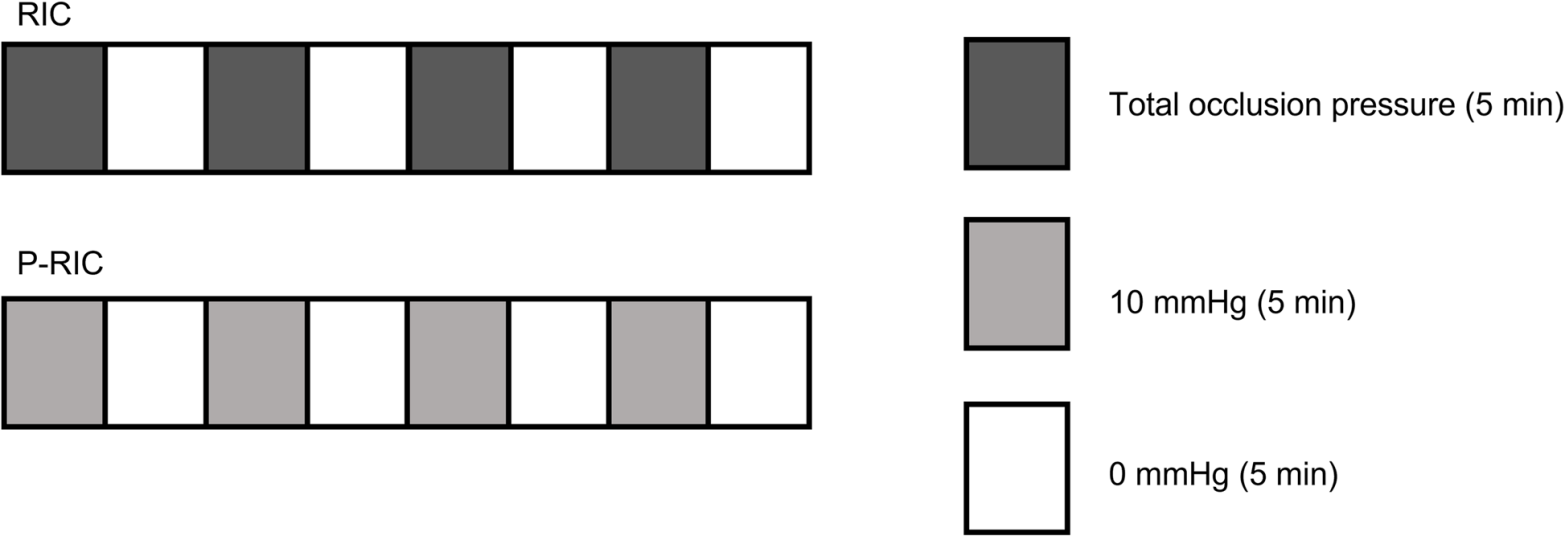
RIC and P-RIC protocol

The RIC protocol will be applied to the proximal region of the thigh of the limb with knee osteoarthritis and if both knees are affected, it will be applied to the knee with the greatest pain complaint [33]. Participants will be relaxed and comfortably positioned in the supine position. The same cuff used to determine TOP will be used and the protocol will consist of four cycles of total ischemia (according to the TOP value determined individually) of 5 min, followed immediately by four cycles of 5 min of vascular reperfusion (0 mmHg), totaling 40 min as shown in Fig. 3.

### Placebo remote ischemic conditioning

The placebo RIC protocol will also be performed on the lower limb with knee osteoarthritis and if both knees are affected, it will be applied on the knee with the greatest pain complaint. Participants will perform a protocol similar to that of RIC, but during the four cycles of 5 min of occlusion, the cuffs will be inflated only with 10 mmHg so as not to cause arterial or venous occlusion alternating with four cycles of 5 min of reperfusion (0 mmHg) (see Fig. 3) [50, 51].

### Outcomes

#### Conditioned pain modulation

CPM is a dynamic measure capable of evaluating endogenous pain inhibition using a “pain-by-pain inhibition” model in which pain in a local area (test stimulus) is inhibited by a second concomitant pain (conditioning stimulus) [3, 52]. In this study, the test stimulus will be performed through the pressure pain threshold using the algometer, and the conditioning stimulus will be performed through the immersion of the hand in cold water, using the cold pressor test (CPT).

First, the markings of the places where the test stimuli will be performed on the participant will be carried out. The participant will be in dorsal decubitus with approximately 20° of hip and knee flexion with the lower limb comfortably supported and markings will be performed in eight locations on both knees: site 1: 2 cm distal to the lower medial border of the patella; site 2: 2 cm distal to the lower lateral border of the patella; site 3: 3 cm lateral to the midpoint on the lateral edge of the patella; site 4: 2 cm proximal to the upper lateral border of the patella; site 5: 2 cm proximal to the upper edge of the patella; site 6: 2 cm proximal to the upper medial border of the patella; site 7: 3 cm medial to the midpoint on the medial border of the patella; site 8: in the center of the patella [53, 54] and in the joint space between the first and second metacarpophalangeal joints (thenar eminence region of the hand) on the contralateral side [33].

These marked sites will receive the test stimulus through the pressure pain threshold test using a pressure algometer which is a reliable and validated instrument (FDX 50/220; Wagner instruments, Greenwich, Connecticut, USA) which has a tip of 1 cm^2^ [55].

The evaluator will apply pressure perpendicularly to the participant’s skin until she reports the change from pressure sensation to painful sensation [33]. The procedure will be performed three times at each site and the mean values in kgf/cm^2^ will be calculated to determine the pressure pain threshold. An interval of 20 s will be maintained between applications to avoid sensitization of pain responses [33].

After each measurement of the pressure pain threshold, pain will be asked using a visual analog scale (VAS) that ranges from 0 (no pain) to 100 (worst imaginable pain). VAS will also be used to measure knee pain at rest and during CPT [5, 33, 54].

After baseline pressure pain threshold measurements, the CPT (conditioning stimulus) [5, 33, 56] will be applied. Participants will be asked to dip their hands in a container of cold water and ice for up to 1 min. The temperature will be monitored by a thermometer (model 5130, Incoterm) and maintained between 1°C and 4°C [33]. Soon after, the pressure pain threshold measurements (test stimulus) will be performed in the same places.

**Fig. 4:**
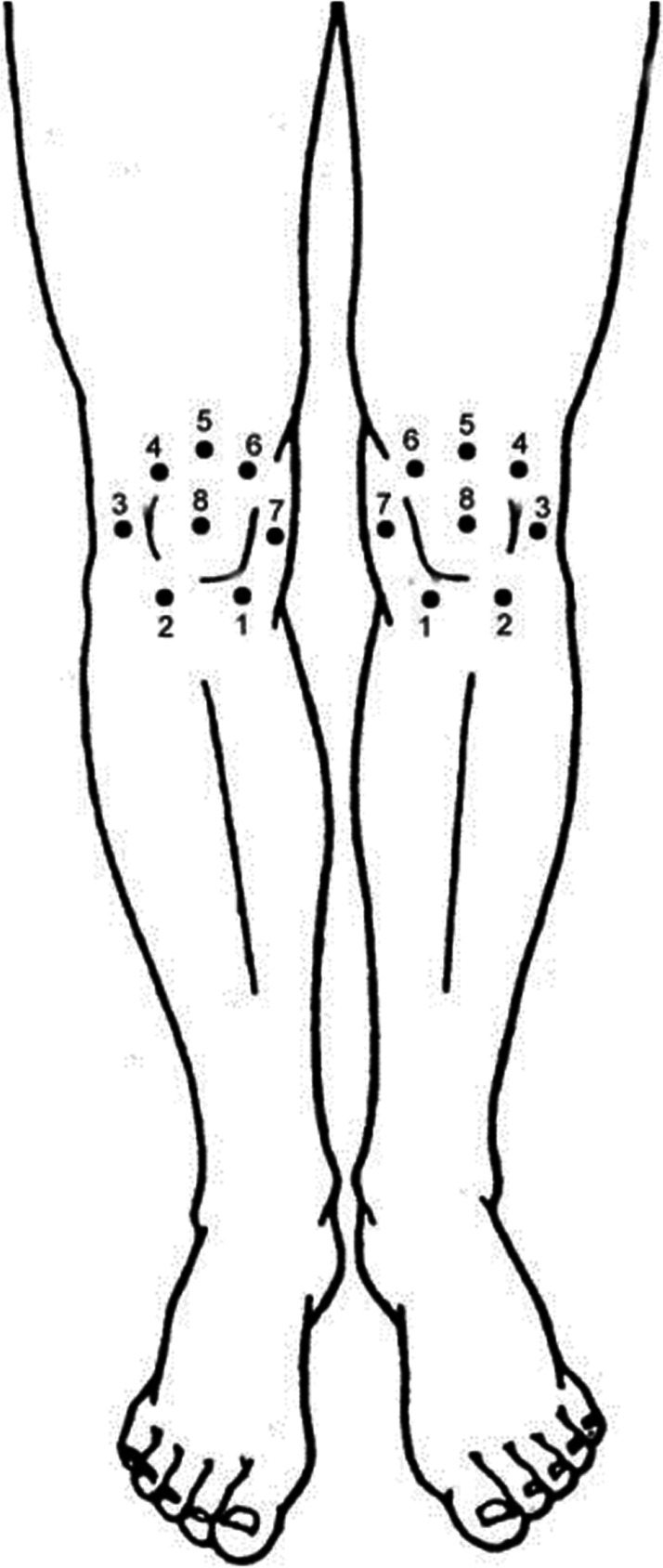
Pressure pain threshold measurement sites [53]

MCD will be quantified by subtracting the second mean pressure pain threshold value (after CPT) from the first mean pressure pain threshold value (before CPT) [CPM = baseline pressure pain threshold − pressure pain threshold Final]. Thus, negative CPM values mean CPM inhibitory activity (higher pressure pain threshold after CPT), while positive values indicate CPM facilitating activity (lower pressure pain threshold after CPT) [3, 33, 56]. The percentage of change will also be calculated using the following formula: [(pain threshold at final pressure − pain threshold at baseline pressure)/pain threshold at final pressure) × 100]. This entire protocol will be repeated after the RIC (Fig. 4) [53].

#### Cardiac autonomic modulation

The collection of data will be carried out individually and the participants will be instructed to remain silent, awake, at rest, and in the supine position. The movement of people on site will be restricted, in order to reduce interference during collection. Participants will be instructed not to consume ANS stimulant substances such as alcoholic beverages, coffee, tea, soft drinks, and chocolate drinks during the 24 h prior to capturing the RR intervals.

The assessment of cardiac autonomic modulation will be performed through HRV by capturing the RR intervals using the heart rate monitor (Polar Electro Oy, Kempele, Finland – model V800) which is a validated equipment [57].

Indices obtained through linear methods, in the time domain (Mean RR, SDNN, and rMSSD), frequency (LF and HF), and the Poincaré plot (SD1 and SD2) will be analyzed [58, 59]. In addition, RR intervals will be analyzed to obtain the 30s rMSSD that represents vagal reentry. All HRV indices will be obtained using the software Kubios HRV – version 3.2 (The Biomedical Signal Analysis Group, Department of Applied Physics, University of Kuopio, Finland) [60].

For this analysis, the time series of RR intervals will be initially submitted to a digital filtering moderated by the software Kubios HRV – version 3.2 [60] complemented by manual filtering, to eliminate premature ectopic beats and artifacts, and only series with more than 95% of sinus beats will be included in the study [58]. Through the visual analysis of the time series, the absence of artifacts or ectopic beats that may interfere with the HRV analysis will be observed.

The series of RR intervals will be analyzed in two different moments: (i) during the performance of the CPM tests and (ii) during the evaluation of the RIC. In order to obtain these analyses, on the first day of the intervention, the following moments will be analyzed: M1 (10 min basal at rest before TOP), M2 (test stimuli through the algometer), M3 (conditioning stimulus, through the CPT), M4 (test stimuli through the algometer), M5 (during RIC), M6 (test stimuli through the algometer), M7 (conditioning stimulus, through the CPT), M8 (test stimuli through the algometer). In these stretches, 256 consecutive RR intervals [59] will be obtained. It is worth noting that at moments M3 and M7, only vagal reentry with a rMSSD of 30 s will be analyzed due to the short time to capture the RR intervals.

On the second day of the intervention, the Pre-RIC (15 min baseline before RIC), RIC (during RIC), and Post-RIC (15 min after RIC) moments will be analyzed, with 1000 consecutive RR intervals being obtained for this analysis [59].

##### Time domain

For analysis of HRV in the time domain, the Mean RR, SDNN, and rMSSD [59–62] indices will be used. The Mean RR index corresponds to the means of all RR intervals [58, 59]. The SDNN index represents the standard deviation of all normal RR intervals recorded in a time interval, expressed in milliseconds (ms), and is considered a measure of global variability, that is, it evaluates both sympathetic and parasympathetic modulation [58, 59]. The rMSSD index corresponds to the root mean square of the differences between adjacent normal RR intervals in a time interval expressed in ms and assesses the behavior of parasympathetic autonomic modulation [6, 58].

##### Frequency domain

In the frequency domain, the following will be used: the low-frequency spectral component (LF — frequency between 0.04 and 0.15 Hz) index that evaluates the sympathetic and parasympathetic behavior, with sympathetic predominance and the high-frequency component (HF — frequency between 0.15 and 0.4 Hz), which is related as a marker of vagal modulation, in normalized units (nu) and in milliseconds squared (ms^2^) [58, 59]. The spectral indices will be calculated from a tachogram using the fast Fourier transform (FFT) algorithm [58, 59].

##### Poincaré plot

The Poincaré plot indices, SD1 and SD2, will be used for a quantitative analysis [59]. The Poincaré plot represents a time series within a Cartesian plane which allows each RR interval to be correlated with the previous interval. It is a geometric method for analysis in which each point is represented on the *x*-axis (horizontal/abscissa) by the preceding normal RR interval, and on the *y*-axis (vertical/ordinate) by the following RR interval [58, 59]. SD1 represents the dispersion of points perpendicular to the line of identity and appears to be an index of instantaneous recording of beat-to-beat variability, representing vagal modulation [58, 59]. SD2, on the other hand, represents the dispersion of points along the identity line and represents the HRV in long-term records, considered a marker of sympathetic and parasympathetic modulation [58, 59, 63].

##### Vagal reentry

Series of RR intervals of the last 30 s of all moments (M1–M11) will be selected in order to compare the moments and assess vagal reentry, as observed in the studies by Imai et al. [64] and Goldberger et al. [65].

### Sample size

The sample size calculation was based on the study by Schliessbach et al. [66], using the CPM test variable. The software G*Power 3.1.9.4 and a reference value of 98 ± 103 kPa were used, using a two-tailed hypothesis test with a test power of 80% and a significance level of 5%. Eventually, 19 participants per group were stipulated. A possible sample loss of 15% was considered so three participants will be added to each group, totaling 44 individuals divided into two groups (22 participants per group).

### Statistical analysis

Statistical analysis will be conducted using SPSS software (version 18; SPSS Inc. Chicago, IL, USA). Data normality will be verified by the Kolmogorov–Smirnov test, if normality is detected, the sample characterization variables will be presented as mean and standard deviation/ if not, in median and interquartile range. To compare the characterization of the sample, Student’s *t* test will be used for independent samples.

Descriptive data of outcomes will be presented in mean, standard deviation, and confidence interval values. Comparisons of outcomes between the groups studied and the moments will be performed using Generalized Linear Mixed Models adjusted to the data with Bonferroni post-test when the main analyzes are significant. In addition, the effects of the groups studied will be verified for all outcomes evaluated by calculating the effect size (ES) through Cohen’s *d*, which will be considered as “null” (< 0.2), “small” (≥ 0.2), “moderate” (≥ 0.6), “large” (≥ 1.2) or “very large” (≥ 2.0) [67]. Intention to treat will be used and, for data imputation, a mixed linear model will be used.

To correlate CPM and cardiac autonomic modulation, the Pearson or Spearman correlation test will be used according to the normality of the data. According to the parameters of Portney and Watkins [68], the correlation magnitude of 0.00 to 0.19 is considered to be very weak; 0.20 to 0.39 weak correlation; 0.40 to 0.69 moderate correlation; 0.70 to 0.89 strong correlation and 0.90 to 1.00 very strong correlation [68]. All analyses will assume a significance level of *p* < 0.05.

The external evaluator will enter the data into the database for screening, randomization, and statistical analysis. Dual data entry in electronic format will be used. Data integrity will be monitored regularly by examining data files for omissions and errors. Participants will receive an anonymous study ID to protect confidentiality, and only study investigators will have access to the final study dataset. The spreadsheets containing the raw numerical data of the data generated in this study will be stored in their entirety initially on two external hard drives and two online clouds. Any data needed to support the protocol can be provided upon request.

### Adverse event reporting and harms

Monitoring all the described variables minimizes any risk of serious injury or cardiovascular complications during RIC, i.e., if the participant experiences sensations such as dizziness, pallor, intense sweating, excessive blood pressure increase, pain, or any signs or symptoms, the procedure will be stopped immediately. Pressure from the cuff in the thigh region can generate discomfort and a feeling of tightness, but these symptoms disappear when the pressure is removed and as described above, all necessary care will be taken. All these variables will be noted in the data collection. Participants who are harmed and require care during or after the study will be referred to the national health service.

## Discussion

### Potential impact and significance of the study

This project can be considered innovative as it is one of the first to investigate the effect of RIC in a chronic musculoskeletal condition and how it affects the cardiac and conditioned autonomic modulation systems of pain. It is believed that the results are able to show a new perspective for the interaction between pain processing and cardiovascular systems; to show the behavior of cardiac autonomic modulation in women with osteoarthritis, since no study on this topic was found in the literature; to discover the acute effect of RIC on pain and on CPM and cardiac autonomic modulation systems.

### Strengths and weaknesses of the study

In addition to not finding any studies in the literature that show a new perspective for the interaction between pain processing and cardiovascular systems in women with knee osteoarthritis, this study has other strengths, which are the high methodological quality of the study. by prospective registration, randomization, restriction of patients and evaluators, intention-to-treat approach, and comparison between a protocol of real ischemic conditioning with a placebo group, allowing to observe the result found in the intervention. We consider it as a weakness or non-blinding of the therapist.

### Contribution and clinical applicability

The present study may provide the therapist and patient with better conditions to ensure greater cardiovascular safety in the use of the intervention. In addition, through these findings, it is intended to trigger knowledge of an acute response for future chronic intervention strategies and that aims to be used in the clinical environment as another strategy that can help, within the multimodal approach, for the management of people with knee osteoarthritis.

It is worth noting that the results will be published in periodicals with a selective editorial policy and presented in conferences. In addition, we will develop a disclosure policy and discuss presentations and outreach with relevant patient and clinical interest groups.

## Trial status

Number Protocol: NCT05059652.

Patient not yet recruitment currently.

Study start date: August 2023.

Primary completion date: December 2023.

Study Completion date: July 2024.

### Supplementary Information

**Additional file 1. **SPIRIT 2013 checklist.

**Additional file 2. **Consent form template.

**Additional file 3.** Protective measures against covid-19.

## Data Availability

The spreadsheets containing the raw numeric data will be stored on two external hard drives and two “clouds.” After analyzing the data, scientific articles related to the study will be made, and after publication, the data will remain preserved and will also be shared and made available in its entirety for a period of 5 years. The datasets analyzed during the current study are available from the corresponding author on reasonable request.

## Abbreviations

CPT: Cold press test
FFT: Fast Fourier transform
HF: High-frequency spectral component
BMI: Body mass index
KOOS: Knee Injury and Osteoarthritis Outcome Score
LF: Low-frequency spectral component
M: Moment
CPM: Conditioned pain modulation
CPM1: Initial conditioned pain modulation
CPM2: Final conditioned pain modulation
Mean RR: Mean of RR intervals
nu: Normalized units
BP: Blood pressure
TOP: Total occlusion pressure
rMSSD: Index that corresponds to the square root of the mean square of the differences between adjacent normal RR intervals in a time interval expressed in milliseconds
RR: Beat-to-beat heart rate
SD1: Instantaneous beat-to-beat variability index
SD2: Index represents HRV in long-term records
SDM: Mean and standard deviation
SDNN: Index representing the standard deviation of all normal RR intervals recorded in a time interval, expressed in milliseconds
ANS: Autonomic nervous system
CNS: Central nervous system
HRV: Heart rate variability
RIC: Remote ischemic conditioning
