# Supplementary material for: Effects of remote ischemic conditioning on conditioned pain modulation and cardiac autonomic modulation in women with knee osteoarthritis: placebo-controlled randomized clinical trial protocol
Source: Trials. 2023 Aug 7;24:502. doi: 10.1186/s13063-023-07527-2 (PMC10405415; doi:10.1186/s13063-023-07527-2)
Supplement: Supplementary file 2 — Additional file 2. Consent form template. [file 13063_2023_7527_MOESM2_ESM.doc]

#

# TERMS OF FREE AND CLEAR CONSENT

**Research Title:** “Effects of remote ischemic conditioning on conditioned pain modulation and cardiac autonomic modulation in women with knee osteoarthritis: placebo-controlled randomized clinical trial protocol”.

**Researcher:** MSc. Taíse Mendes Biral Duarte.

**Advisor:** PhD. Professor Franciele Marques Vanderlei.

The information contained in this form, provided by FRANCIELE MARQUES VANDERLEI and TAÍSE MENDES BIRAL DUARTE, aims to invite the volunteer to participate in the research mentioned above, authorizing his participation with full knowledge of the nature of the procedures to which he will be submitted.

1. **Nature of the research:** You are being invited to participate in this research that aims to analyze and compare the effects of ischemic conditioning on pain and cardiac autonomic control systems. Ischemic conditioning consists of cycles where a manometer is used, which will apply pressure on the thigh for five minutes, then it will be performed for five minutes without pressure, alternating four times each.
2. **Research participants:** There will be 44 women aged 50 to 80 with knee osteoarthritis. To be part of this group you cannot be a smoker (the act of consuming cigarettes or other products containing tobacco, whose component is nicotine), use medication that stimulates the heartbeat, drink alcohol or any other type of illegal drug. In addition, you will be included in the study if you have unilateral or bilateral knee osteoarthritis [34–36] diagnosed according to American College of Rheumatology criteria [37] which are: criteria for clinical and radiographic diagnosis (having knee pain, osteophytes and having at least one of the three items—age greater than 50 years; stiff- ness lasting less than 30 min and/or crepitus) or criteria for clinical diagnosis (pain in the knee and having at least three of the six items — age greater than 50 years; stiff- ness lasting less than 30 min; crepitus; bone enlargement; bone tenderness; no palpable heat)a medical diagnosis of osteoarthritis of one or both knees.
3. **Research Involvement:** By participating in this study, you will allow the researcher to carry out a physical examination and the application of a questionnaire, containing her personal data and assessing her general state of health, medications she uses.

You will be instructed not to perform vigorous physical exercise 24 hours before the days of data collection and to have a light meal at least two hours before the procedures.

You will attend the clinic on two days separated by a week. Initially, you will be evaluated for anthropometric characteristics, using a scale to measure body weight and a stadiometer, which is a device for measuring height. Your blood pressure will also be measured using a sphygmomanometer and then a questionnaire will be applied that asks about your pain, other symptoms, activities of daily living, role in sport, leisure and quality of life related to the knee.

After these initial procedures, measures of blood flow restriction will be evaluated, which is the assessment of the amount of pressure needed to safely restrict blood flow without causing harm. This procedure will be carried out using a pressure cuff that will be inflated in the thigh and will provide a feeling of tightness. Then, the initial assessment of the conditioned modulation of pain will be performed using a pressure algometer, which is a device that will apply pressure to the skin to measure your pain threshold, followed by a test immersion of your hand in cold water and again the threshold from pain to pressure with the algometer. Muscle pain will be assessed using a visual analogue scale to quantify your pain from 0 to 100. Cardiac autonomic control will be assessed using a cardiofrequency meter attached to your chest.

Then, you will carry out the referred ischemic conditioning protocol that lasts 40 minutes where the presence of pressure in the thigh is alternated and its absence. After this protocol, the initial evaluation procedures described above will be evaluated again.

On the second day, the assessment of cardiac autonomic modulation will be performed again with the heart rate monitor, followed by the intervention with conditioning and, finally, the assessment of cardiac autonomic modulation.

1. **About the interviews:** The sessions will be held at the Sports Physiotherapy Laboratory of the São Paulo State University (Unesp) in a room with controlled temperature and humidity environments, located at 305 Roberto Simonsen Street, Educational Center - Presidente Prudente, SP, depending on the participant's availability. The times will be previously scheduled and controlled by the person responsible for the research. The assessments, initial and final, will be scheduled in advance and will also be held at Sports Physiotherapy Laboratory.
2. **Risks and discomfort:** The procedures adopted in this research comply with the Ethics Criteria for Research with Human Beings according to Resolution nº 466/12 of the National Health Council. None of the procedures used pose a risk to her dignity. Monitoring all the described variables minimizes any risk of serious injuries or cardiovascular complications during conditioning, that is, if you feel sensations such as dizziness, pallor, intense sweating, excessive increase in blood pressure, pain or any signs or symptoms, the procedure will be stopped immediately. Pressure from the cuff in the thigh region can cause discomfort and a feeling of tightness, but these symptoms disappear when the pressure is removed and, as described above, all the necessary care will be taken.
3. **Confidentiality about the Identity of Research Subjects:** Your identity, as well as information or any other means that may identify you, will be kept confidential. Only the researcher and her advisor (and/or research team) will be aware of her identity and we undertake to keep it confidential when publishing the results of this research.
4. **Data Confidentiality:** The information collected in this study that is not published in the research will not be disclosed in any other way and the documents containing such information will be destroyed in accordance with the current Regulations of National Commission of Ethics in Research.
5. **Benefits:** By participating in this research you will not have any direct benefit. However, we hope that this study will provide important information about the effects of ischemic conditioning on conditioned pain modulation and cardiac autonomic control in women with knee osteoarthritis who experience pain. In addition, we hope that the knowledge that will be built from this research can be used for the benefit of other people who also have osteoarthritis and can contribute to advances in rehabilitation processes. The researchers involved in the research are committed to disclosing the results obtained, respecting the secrecy of the information collected, as provided for in the previous item.
6. **Payment:** You will not incur any expenses to participate in this research, and nothing will be paid for your participation.

You are free to refuse to participate and even refuse to continue participating in any phase of the research, without any prejudice to you. Whenever you want, you can ask for more information about the research through the project researcher's telephone number and, if necessary, through the Research Ethics Committee's telephone number.

After these clarifications, we ask for your free consent to participate in this research. Therefore, please complete the following items: I confirm that I have received a copy of this consent form, and I authorize the execution of the research work and the disclosure of the data obtained in this study.

Note: Do not sign this term if you still have questions about it.

# Free and Informed Consent

In view of the items presented above, I, in a free and informed manner, express my consent to participate in the research

___________________________

Research Participant Name

______________________________

Research Participant Signature

__________________________________

Researcher Signature

___________________________________

Advisor's Signature

**"The Research Ethics Committees are interdisciplinary and independent collegiate bodies, of public relevance, with an advisory, deliberative and educational nature, created to defend the interests of research participants in their integrity and dignity and to contribute to the development of research within ethical standards. (CNS Resolution 466/12, VII.2 and CNS Resolution 510/16)”**

**Researcher:** MSc. Taíse Mendes Biral Duarte - (18) 99666-3537

**Advisor:** PhD. Professor Franciele Marques Vanderlei - (18) 99709-1656

**Coordinator of the Research Ethics Committee:** PhD. Professor Edna Maria do Carmo

**Deputy Coordinator of the Research Ethics Committee:** PhD. Professor Andreia Cristiane Silva Wiezzel

**Committee Phone:** 3229-5315/ 3229-5526

**E-mail:** [**cep.fct@unesp.br**](mailto:cep.fct@unesp.br)
