# Supplementary material for: Effects of remote ischemic conditioning on conditioned pain modulation and cardiac autonomic modulation in women with knee osteoarthritis: placebo-controlled randomized clinical trial protocol
Source: Trials. 2023 Aug 7;24:502. doi: 10.1186/s13063-023-07527-2 (PMC10405415; doi:10.1186/s13063-023-07527-2)
Supplement: Supplementary file 3 — Additional file 3. Protective measures against covid-19. [file 13063_2023_7527_MOESM3_ESM.docx]

**Protective measures against covid-19**

For the safety of the participants and evaluators involved, protective measures will be taken against COVID-19: social distancing, evaluation of only one participant will be carried out at a time, and only the strictly necessary staff will remain on site. In addition, gel alcohol will be made available for participants and for the hygiene of equipment and the use of face masks will be mandatory for all present on site.

As a protective measure, participants will be asked over the phone about COVID-19 specific symptoms, such as tiredness, cough, sore throat, runny nose, fever, loss of smell or taste buds) on the day before they arrive at the collection site to ensure greater safety and if symptoms are present, they will be instructed to seek the nearest health center or hospital for immediate care ^(1)^.

1. Governo Federal: Ministério da Saúde [Internet]. [place unknown]; 2021 Apr 08. Sintomas; [cited 2021 Oct 5]; Available from: https://www.gov.br/saude/pt-br/coronavirus/sintomas.
